# Minority and Majority Adolescents’ Attitudes toward Mutual Acculturation and its Association with Psychological Adjustment

**DOI:** 10.1007/s10964-022-01604-6

**Published:** 2022-04-06

**Authors:** Petra Sidler, Gülseli Baysu, Wassilis Kassis, Clarissa Janousch, Raia Chouvati, Christos Govaris, Ulrike Graf, Christian Rietz

**Affiliations:** 1 Institute for Research and Development, School of Education, University of Applied Sciences and Arts Northwestern Switzerland, Brugg-Windisch, Switzerland; 2 School of Psychology, Queen’s University, Belfast, United Kingdom; 3 Department of Primary Education, University of Thessaly, Thessaly, Greece; 4 Faculty of Educational and Social Sciences, University of Education Heidelberg, Heidelberg, Germany

**Keywords:** Mutual acculturation, Majority acculturation, Psychological adjustment, Self-esteem, Self-determination, Latent profile analysis

## Abstract

Although acculturation is considered a mutual process, no measure assesses attitudes toward mutual acculturation. Through a novel four-dimensional measurement, this study addresses this research gap by assessing attitudes toward minority and majority acculturation and its relation to psychological adjustment for immigrant-background minority and non-immigrant majority adolescents in public secondary schools in three European countries: in Germany (*n* = 346, 46% female, *M*_age_ = 12.78 years, range 11–16), Greece (*n* = 439, 56% female, *M*_age_ = 12.29 years, range 11–20), and Switzerland (*n* = 375, 47% female, *M*_age_ = 12.67 years, range 11–15). Latent profile analyses led to three distinct acculturation profiles in all three countries: strong and mild mutual integration profiles, where both migrant and majority students are expected to integrate, and a third profile assuming lower responsibility upon the majority. Additionally, those in the strong- and mild-integration profiles reported stronger psychological adjustment than those assuming lower responsibility upon the majority, which held for all students in Switzerland and mostly for those without a migration background in Germany. The findings demonstrate the importance of a mutual acculturation framework for future research. Moreover, as most adolescents fit in with one of the mutual integration patterns, findings stress that no matter their migration background, adolescents favor mutual integration including the expectation on schools to enhance intercultural contact.

## Introduction

Societies and schools are becoming increasingly more culturally diverse. With such diversity comes several challenges as members of different groups, including the youth, acculturate and learn to mutually accommodate each other. Acculturation is defined as the cultural and psychological change of individuals and groups when having intercultural contact (Berry, 2019), which implies that both groups can change. However, acculturation attitudes are commonly assessed by combining attitudes toward only *minority group members’* (a) maintenance of their heritage culture and (b) dominant culture adoption (Bourhis et al., 1997). Following theoretical considerations on the reciprocity and mutuality of acculturation (Chirkov, 2009), empirical research has started assessing not only minority but also majority acculturation (Kunst et al., 2021). However, assessing attitudes toward both minority and majority acculturation is still a research gap. Furthermore, in the school context, migrant students’ acculturation has often been analyzed in relation to their adjustment, finding an integration strategy to be predominantly positively associated with school and psychological adjustment, yet also stressing inconsistent results due to a variety of contexts and assessments (Makarova & Birman, 2016). However, this research relies heavily on measures of minority group members’ acculturation, thus understanding the relation of attitudes toward mutual acculturation and adjustment presents another research gap. To address these gaps, a novel four-dimensional mutual acculturation attitudes scale was used, which considers both immigrant-background minority and non-immigrant-background majority adolescents’ attitudes toward mutual acculturation—that is, their attitudes toward *migration background students’ heritage culture maintenance* and *dominant culture adoption*, as well as toward *majority students’ acquisition of cultural knowledge* and *schools’ endorsement of intercultural contact* (Sidler et al., 2021). Thus, to address the first research gap, attitudes toward mutual acculturation of both, immigrant-background and non-immigrant-background youth are analyzed and, to address the second research gap, put into relation with psychological adjustment.

### Mutual Acculturation at School

In the tradition of cross-cultural psychology, acculturation results from intercultural contact and is a process of continuous cultural and psychological change (Berry, 2019). The words *ad cultura* are Latin for “leading to a culture” (Zick, 2010). In this study, culture is understood as a vague, dynamic, and always-changing concept (MacLachlan et al., 2004), which helps to make sense of the historically diverse conceptualizations and assessments of acculturation. Culture includes artefacts, behaviors, and attitudes or values (Rudmin, 2009). Thus, acculturation is understood as the process of dealing with and adjusting to a change in visible artefacts, behaviors, and attitudes within a specific context, which shapes the relationship between acculturation and adjustment (Birman & Simon, 2014). Following the bidimensional assessment of acculturation attitudes (Bourhis et al., 1997), the combination of the two minority dimensions (heritage culture maintenance and dominant culture adoption) leads to four acculturation strategies: *integration* (agreement with both dimensions), *assimilation* (agreement with adoption, disagreement with maintenance), *separation* (agreement with maintenance, disagreement with adoption), and *marginalization/individualism* (disagreement with both dimensions).

The school context is an acculturation context and is acknowledged to be a key environment for adolescents’ continued acculturation (Horenczyk & Tatar, 2012). Whether and how schools handle cultural diversity affects belonging and educational outcomes of both, migrant and majority students, as acculturation affects both groups (Baysu et al., 2021). Thus, acculturation is defined as a mutual process, and members of the majority group and minority groups are *acculturating agents* (Sidler et al., 2021). This contrasts with how majority acculturation commonly has been assessed. The interactive acculturation model (Bourhis et al., 1997), for example, assessed majority and minority attitudes toward minority acculturation. In this sense, majority acculturation would relate to majority members’ attitudes toward minority acculturation. Similarly, more recent research assessing majority acculturation attitudes or expectations in the school context has measured majority members’ attitudes toward minority acculturation, therefore focusing on the minority group members as the only acculturating agents (Makarova & Birman, 2015, 2016). However, an interactive acculturation model, in the sense that both sides are interacting and adjusting, relates not only to the two different perspectives at stake (minority and majority adolescents having attitudes toward minority acculturation) but to who is experiencing a change in the cultural context and, thus, who is acculturating (minority and majority adolescents having attitudes toward minority and majority acculturation, the latter including schools). In diverse schools, peer interactions lead to contact with a variety of cultural backgrounds (Miklikowska, 2017); thus, not only immigrant-background but also non-immigrant-background students experience intercultural contact and therefore acculturation at school.

Moreover, intercultural interactions and communications require minority and majority students to acquire new intercultural skills (Landis & Bhawuk, 2020), meaning that both, non-immigrant background, and immigrant background adolescents adjust to the intercultural context at school and therefore acculturate. Yet, national institutions like schools must also be adapted to better satisfy the needs of all groups and individuals living together (Berry, 2019). Thus, next to being an acculturation context, schools are cultural actors with pervasive power ideologies (Warikoo & Carter, 2009). Schools also have agency insofar that they actively shape the setting of intercultural contact and intercultural learning by implementing school diversity policies (Celeste et al., 2019), raising awareness about implicit bias and stereotypes (Warikoo et al., 2016), or ignoring the facticity of intercultural contact. Diversity approaches at school were found to affect ethnic minority and majority adolescents (Baysu et al., 2021), illustrating schools’ agency as well as how immigrant-background and non-immigrant-background students are affected by it. Majority-group members and institutions thus become recipients and minority-group members become agents of social change (Kunst et al., 2021). Therefore, instead of focusing only on minority students as acculturating agents, majority students and schools are also considered in this study.

To clarify, in this study, the term *minority acculturation* relates to the acculturation of those who have a migration background (including the first, second, and 2.5 generation), and *majority acculturation* relates to the acculturation of those who do not have a migration background (the so-called majority members or natives). Thus, minority acculturation and minority or migrant students relate to students with a migration background, whereas majority acculturation and majority students relate to students without a migration background.

### Acculturation and Psychological Adjustment

In the school context, three aspects of adolescents’ adjustment are commonly measured as acculturation outcomes: a) students’ psychological adjustment including their self-esteem and self-determination; b) students’ sociocultural adjustment such as acquiring school-relevant competence and conduct as well as having good social relationships with other students and teachers; and c) students’ educational outcomes and aspirations (Makarova & Birman, 2015, 2016). This study focuses on two indicators of psychological adjustment, namely self-esteem and self-determination, as important components of adolescent adjustment in school. Empirical research on the associations between acculturation and different components of adolescent adjustment has yielded inconsistent results (Makarova & Birman, 2015, 2016). For adolescents with a migration background, an integration strategy is often associated with successful adjustment at school (Nguyen & Benet-Martínez, 2013); however, it often also introduces challenges to adolescents with a migration background (Brown et al., 2013). Moreover, an assimilation strategy was also found to lead to positive adjustment, particularly in contexts expecting assimilation (Makarova & Birman, 2015). Thus, the specific school setting plays an important role when it comes to the association of acculturation and psychological adjustment for adolescents with a migration background.

Research on majority acculturation and adjustment is scarce, yet a study in Norway assessed majority students’ culture maintenance and their adoption of immigrants’ cultures (Haugen & Kunst, 2017). They found that separated majority members reported more identity threat, more ethnic discrimination, and higher self-esteem than integrated and undifferentiated majority members. In another study, it was found that majority members’ openness predicted more adoption of minority cultures, whereas conscientiousness predicted less adoption of the minority cultures (Kunst et al., 2021). As intergroup contact experiences and cross-group friendships were found to be beneficial for everyone (Killen et al., 2007) and as cultural diversity is increasing in schools, majority students’ acculturation may also be related to their psychological adjustment.

It should be acknowledged that most studies assessing the relation of acculturation and adjustment, including this study, are cross-sectional, questioning the direction of association between acculturation and adjustment (Kunst, 2021). Moreover, those who did assess the association longitudinally found little evidence for the integration hypothesis, meaning that an integration orientation is most conducive to adjustment (Bierwiaczonek & Kunst, 2021). However, the absence of longitudinal associations, which often control for cross-sectional ones, does not undermine the presence or importance of cross-sectional associations. Thus, while assessing how acculturation is interrelated to psychological adjustment, this study does not imply causality (Grigoryev & Berry, 2022), as the associations can work both ways.

### Geographical Contexts: Germany, Greece, and Switzerland

From an ecological perspective (Bronfenbrenner, 1979), schools are embedded in and can interact with national contexts as distal contexts that have integration policies considering immigrant minorities. Nation-states and their climate, policies, and schools are important macro contexts for adolescents’ acculturation (Motti-Stefanidi et al., 2012). Acculturation attitudes and ethnic identities may vary relative to the country of residence and may be linked to multicultural climate and policies (Yağmur & Van de Vijver, 2012).

Germany, Greece, and Switzerland are interesting case studies because of their partly overlapping and partly dissimilar migration histories and integration policies toward immigrants (Migrant Integration Policy Index [MIPEX], 2020). Germany and Switzerland are commonly considered typical migration-arrival countries, with Germany having about 13,380,000 (16% of the total population) and Switzerland having about 2,550,000 (30% of the total population) foreign-born residents in 2019 (OECD, 2021). Concerning Greece, whereas many people moved through Greece onwards to other European countries, many also remained: In 2019, the foreign-born population in Greece amounted to 1,340,000 (13% of the total population; OECD, 2021). In terms of integration policies, the MIPEX assesses policies for integrating migrants to create a multidimensional picture of equal rights and migrants’ opportunities to participate in society. Concerning education, the MIPEX captures how accessible education is for migrant students, how teachers are being trained in dealing with cultural diversity at school, and whether migrant students’ special needs are considered. In 2019, Greece scored 46 (unfavorable political participation and slightly unfavorable education and access to nationality), Switzerland scored 50 (slightly unfavorable anti-discrimination and access to nationality), and Germany scored 58 points (no unfavorable ratings). It is thus explored whether the prevalence and effectiveness of integration profiles would differ across these three countries ranging from more to less unfavorable attitudes toward diversity.

## The Present Study

This study assessed immigrant background and nonimmigrant background adolescents’ attitudes toward mutual acculturation, and the associations between their acculturation profiles and psychological adjustment. Following previous research, this study expected to find the so-called integration profiles as the most common profiles in each country and that these integration profiles should predict psychological adjustment for students with and without a migration background. Primarily, this analysis offers cross-national insights into adolescents’ attitudes toward mutual acculturation, including minority and majority acculturation in three ways: First, minority and majority students and schools are defined as acculturating agents and included in the measurement. Then, the attitudes of students with and without a migration background are analyzed in each country, taking their specific perspective on mutual acculturation into account, thus resulting in various acculturation profiles. Finally, a person-centric approach is employed to analyze adolescents’ endorsement of four acculturation dimensions, which allows for identifying acculturation profiles inductively, rather than assuming a fixed set of profiles or groups, as is usually done in the acculturation framework. The combination of these three points is a novel approach and enhances acculturation research. Additionally, building upon the acculturation profiles leads to cross-national insights into their relations with psychological adjustment like self-esteem and self-determination. Thus, the aim of this study was twofold: first, to explore adolescents’ attitudes toward mutual acculturation, and second, to assess the relation between these acculturation profiles and psychological adjustment.

## Methods

### Participants

Participants were part of a random convenient sample of seventh graders from lower secondary education classes (like middle school in the United States) in rural and urban regions of Germany, Greece, and Switzerland. The German sample comprised 346 students in 14 schools (46% female, 54% male, 0.3% other, *M*_age_ = 12.78 years, *SD* = 0.78). The Greek sample comprised 439 students in 14 schools (56% female, 44% male, 0.2% other, *M*_age_ = 12.29 years, *SD* = 0.88), and the Swiss sample comprised 375 students in 20 schools (47% female, 53% male, 0% other, *M*_age_ = 12.67 years, *SD* = 0.69).

Given the presence of first-generation immigrant students in each country (Germany: 28% first-generation; Greece: 12% first-generation; Switzerland: 19% first generation), questionnaires were not only prepared in the two national languages (German and Greek) but also translated into five additional languages (Arabic, English, Farsi, French, and Turkish) following the four-eyes principle, a content translation, and a culturally sensitive approach (Peña 2007). Most students in each country filled out the questionnaire in their country’s official language (93 and 96% of the students in German in Germany and Switzerland, respectively; 91% in Greek in Greece).

As migration background is defined diversely in the three countries, migration background was operationalized dichotomously by combining three single-item indicators: country of birth of students and their parents and students’ nationalities. If students and their parents were all locally born (i.e., in Germany, Greece, or Switzerland) and students had only the local (i.e., only the German, Greek, or Swiss) nationality, they were identified as not having a migration background—that is, as belonging to the majority. Otherwise, if any of these conditions were not met, the respective student was identified as having a migration background (79% in Germany, *n* = 272; 47% in Greece, *n* = 207; and 76% in Switzerland, *n* = 283).

### Procedure and Sampling

The data used for this study was collected in 2019 and 2020 through a web-based survey within the project *Overcoming Inequalities with Education: School and Resilience* of the NCCR–On the Move. In all three countries, data collection was guided by research assistants who visited school classes during school time, and students filled out the questionnaire via tablets in approximately 1 h. Research assistants instructed the students and answered possible questions to ensure a similar data collection environment. A protocol was written for each school class. In line with ethics approval in each country, parental consent and child assent were received.

In each country, a local ethical committee was contacted for approval before the sampling started. In Germany, upper-level school offices and school districts were contacted via e-mail and/or telephone. With their permission, teachers of the seventh grade were contacted by e-mail or phone. Teachers informed the students and their parents about the study and asked for consent in writing. In sum, 14 schools with 28 classes were recruited in the two northern upper-level school offices, Karlsruhe and Stuttgart, in the region of Baden-Württemberg. In Greece, schools were contacted in three regions, namely Athens, Larisa, and Crete. Local school counselors advised and helped in recruiting classes. The focus was on schools in Athens, as 34.6% of the Greek student population goes to school in Athens (Hellenic Statistical Authority 2020). Whereas schools in Athens have a multicultural composition, immigrants and refugees in Larisa and on the island of Crete are considered to be more integrated in the local society (Kotoyannos et al. 2019). In total, 14 schools with 48 classes were recruited. In Switzerland, cantonal educational offices were contacted first. Afterwards, school directors and then class teachers were contacted via email and phone calls. Through the teachers, the parents and students were informed and asked for consent. Twenty schools with a total of 32 classes were recruited in the Aargau, Basel-Stadt, and Solothurn cantons.

The sample size was not determined by an a priori power analysis: the three national samples are convenient random samples, meaning that as many schools as possible were contacted to recruit full classes. The Swiss sample (46% female, *n* = 167; 53% Swiss, *n* = 193), consisting of students of the vocational and technical school tracks, was compared with official statistics of students in the relevant cantons. The gender and immigrant composition of the Swiss sample was comparable to cantonal statistics, as the lowest school level has a lower percentage of females and Swiss nationals (FSO, 2020a, 2020b), but in other countries, such population data were not available.

### Measures

#### Attitudes toward mutual acculturation

The four-dimensional assessment of attitudes toward mutual acculturation consists of seven items per dimension (Sidler et al., 2021; see Supplementary Material Table 1B for all items verbatim). Using a 4-point Likert scale ranging from *disagree* (1) to *agree* (4), it measures attitudes toward (1) *Migration background students’ heritage culture maintenance* (e.g., “I find that it is important for teenagers from another country who live in [country] to be allowed to preserve their traditions and customs.”; Germany, Greece, Switzerland Cronbach αs^1^, respectively, 0.85, 0.85, 0.84); (2) *Migration background students’ dominant culture adoption* (e.g., “I find that it is important for teenagers from other countries who live in [country] to adopt the dominant way of life in [country].”; Germany, Greece, Switzerland Cronbach αs respectively 0.88, 0.88, 0.91); (3) *Majority students’ acquisition of cultural knowledge* (e.g., “I find it is important that German/Greek/Swiss teenagers who live in [country] have to get to know the religions of teenagers from other countries who live in [country].”; Germany, Greece, Switzerland Cronbach αs, respectively 0.91, 0.91, 0.92); (4) Schools’ endorsement of intercultural contact (e.g., “I find it is important that the [country] schooling system allows teenagers from other countries and [country] teenagers to exchange information about languages.”; Germany, Greece, Switzerland Cronbach αs, respectively 0.90, 0.90, 0.92).

Higher scores indicate higher agreement with the relevant dimension. Each dimension was calculated through the means of at least one up to all seven items. Both Cronbach’s alphas and McDonald’s omegas showed high reliability across all countries and dimensions, from alpha and omega of 0.84 (migrant students’ heritage culture maintenance in Switzerland) up to 0.92 (majority students’ acquisition of cultural knowledge and schools’ endorsement of intercultural contact in Switzerland).

Given the multi-level structure of the data, intraclass correlations were assessed on two (students – school classes) and three levels (students—school classes—schools). Across all three countries, intraclass correlations were all lower than 0.100, indicating small variance at the school and class level.

Given the cross-national design of this study, confirmatory factor analyses (CFA) were run in each country (see Supplementary Material, Table 2B for factor loadings) and cross-national measurement invariance (see Supplementary Material, Table 3B for multigroup-CFA values) was assessed in JASP (0.16.0.0). The 28-item and four-factor model of the assessment of attitudes toward mutual acculturation was tested and confirmed through a CFA across the three independent samples. Results showed good factor loadings and a sufficient fit across countries (Xia & Yang, 2019). First, assessing the invariance of the factor structure across countries supported the configural invariance (the baseline model, M2 in Table 3B in the Supplementary Material). Comparing the baseline model to the model with constrained factor loadings (M3) supported the metric invariance. Finally, further constraining the intercepts to be equal (M4) supported the scalar invariance (Xia & Yang, 2019). These results show that comparisons across the German, Greek, and Swiss samples are meaningful and valid.

#### Self-esteem

The Rosenberg Self-Esteem Scale (Rosenberg, 1965) is a 10-item scale that assesses global self-worth through positive and negative feelings concerning oneself. Respondents answer on a 4-point Likert scale ranging from *strongly disagree* (1) to *strongly agree* (4; e.g., “Overall, I am satisfied with myself.”). Cronbach’s alpha showed good reliability across all countries, and McDonald’s omega showed good reliability for Germany and Switzerland, however, there was no value for Greece (Germany *α* = 0.78 and *ω* = 0.75; Greece *α* = 0.73 and *ω* = no value; and Switzerland *α* = 0.82 and *ω* = 0.80).

#### Self-determination

General self-determination was assessed following Deci and Ryan’s (2010) self-determination theory adapted to the school context. Respondents answered questions on a 4-point Likert scale ranging from *do not agree* (1) to *agree* (4). The 18 items assess the three basic needs dimensions of autonomy (e.g., “I was free to do things in my own way.”), competence (e.g., “I finished difficult tasks and assignments successfully.”), and relatedness (e.g., “I felt I was very close with and had strong bonds with classmates who are important to me.”). Cronbach’s alpha showed acceptable reliability across all countries, and McDonald’s omega showed acceptable reliability for Greece and Switzerland, however, there was no value for Germany (Germany *α* = 0.65 and *ω* = no value; Greece *α* = 0.70 and *ω* = 0.65; and Switzerland *α* = 0.78 and *ω* = 0.75).

#### Gender

Students reported their gender as either “girl,” “boy,” or “other.” For data analysis, a dummy variable (male = 1, female or other = 0) was used, following theories on dominant masculinities (Connell, 1998).

## Results

### Descriptive Statistics

Table 1 shows the descriptive statistics (means and standard deviations) for the German, Greek, and Swiss samples. The reliabilities of the four acculturation attitudes are good to excellent in all three samples. Students in all three countries agree quite uniformly with immigrant students maintaining their cultural characteristics. Students in all three countries also agree with the two majority dimensions: majority students’ acquisition of cultural knowledge and schools’ endorsement of intercultural contact. The most variety arose in the second dimension, immigrant students adopting the dominant cultural characteristics, with students in Greece tending to agree whereas students in Germany and Switzerland were indecisive. Differences between the three samples concerning the four dimensions were studied using univariate analysis of variance (ANOVA). Statistically significant differences were found in the second dimension, migrant students’ dominant culture adoption, and the fourth dimension, schools’ endorsement of intercultural contact. The Greek sample scored significantly higher on the adoption dimension than the German and Swiss samples, *F*(2, 1043) = 23.35, *p* < 0.001, η^2^ = 0.04. Additionally, the Greek sample scored significantly higher on the school dimension than the German and Swiss samples, *F*(2, 1044) = 4.93, *p* = 0.007, η^2^ = 0.01. No further statistically significant differences were found between the three national samples for the first dimension, migrant students’ heritage culture maintenance, or the third dimension, majority students’ acquisition of cultural knowledge.

**Table 1:** Mean and standard deviation of the four acculturation dimensions and psychological adjustment

	Country	*n*	*M*	*SD*
Migrant students’ heritage culture maintenance	GER	327	3.43	0.65
	GRE	406	3.42	0.68
	SWI	356	3.42	0.60
Migrant students’ dominant culture adoption	GER	308	2.52	0.85
	GRE	395	2.86	0.80
	SWI	343	2.48	0.86
Majority students’ acquisition of cultural knowledge	GER	321	2.97	0.80
	GRE	397	3.08	0.78
	SWI	346	2.96	0.78
Schools’ endorsement of intercultural contact	GER	320	3.13	0.76
	GRE	382	3.28	0.67
	SWI	345	3.14	0.75
Self-esteem	GER	325	2.91	0.55
	GRE	359	3.01	0.51
	SWI	345	2.95	0.54
Self-determination	GER	330	2.82	0.41
	GRE	404	2.83	0.42
	SWI	357	2.90	0.45

The four acculturation dimensions and the two psychological adjustment measures were assessed on a 4-point Likert scale ranging from 1 (totally disagree) to 4 (totally agree)

*GER* Germany, *GRE* Greece, *SWI* Switzerland, *M* mean, *SD* standard deviation

Table 2 shows correlations between the four dimensions and psychological adjustment. Correlations between the four dimensions varied in effect strength across countries, but all correlations were positive in all three countries (and all but three were significant). Correlations between self-esteem and self-determination were significant and positive in all three countries. Correlations between psychological adjustment and the four acculturation dimensions, however, varied greatly in terms of statistical significance as well as the direction and strength of association across dimensions and countries.

**Table 2:** Correlations between the four acculturation dimensions and psychological adjustment (assessed via self-esteem and self-determination)

	Country	Migrant students’ heritage culture maintenance	Migrant students’ dominant culture adoption	Majority students’ acquisition of cultural knowledge	Schools’ endorsement of intercultural contact	Self-esteem
Migrant students’ heritage culture maintenance	GER	1				
	GRE					
	SWI					
Migrant students’ dominant culture adoption	GER	0.118* (*n* = 306)	1			
	GRE	0.097 (*n* = 387)				
	SWI	0.004 (*n* = 343)				
Majority students’ acquisition of cultural knowledge	GER	0.296*** (*n* = 316)	0.405*** (*n* = 302)	1		
	GRE	0.355*** (*n* = 384)	0.347*** (*n* = 382)			
	SWI	0.453*** (*n* = 343)	0.202*** (*n* = 339)			
Schools’ endorsement of intercultural contact	GER	0.333*** (*n* = 311)	0.225*** (*n* = 297)	0.609*** (*n* = 311)		
	GRE	0.409*** (*n* = 369)	0.233*** (*n* = 368)	0.596*** (*n* = 369)	1	
	SWI	0.518*** (*n* = 343)	0.052 (*n* = 338)	0.583*** (*n* = 340)		
	GER	0.215*** (*n* = 317)	0.014 (*n* = 299)	0.110 (*n* = 310)	0.058 (*n* = 312)	
Self-esteem	GRE	0.062 (*n* = 349)	−0.105 (*n* = 343)	−0.036 (*n* = 347)	0.046 (*n* = 338)	1
	SWI	0.219*** (*n* = 342)	−0.035 (*n* = 332)	0.079 (*n* = 336)	0.172** (*n* = 337)	
	GER	0.250*** (*n* = 320)	0.005 (*n* = 303)	0.082 (*n* = 315)	0.108 (*n* = 313)	0.547*** (*n* = 318)
Self-determination	GRE	0.134** (*n* = 390)	−0.083 (*n* = 382)	0.007 (*n* = 379)	0.093 (*n* = 362)	0.575*** (*n* = 343)
	SWI	0.269*** (*n* = 352)	−0.093 (*n* = 342)	0.119* (*n* = 344)	0.210*** (*n* = 343)	0.634*** (*n* = 342)

Missing data was excluded pair-wise

*GER* Germany, *GRE* Greece, *SWI* Switzerland

*p ≤ 0.05; **p ≤ 0.01; ***p ≤ 0.001

### Acculturation Profiles via Latent Profile Analysis

To answer the first research question, latent profile analyses (LPA) were conducted with the four dimensions of the attitudes toward mutual acculturation (migrant students’ heritage culture maintenance, migrant students’ dominant culture adoption, majority students’ acquisition of cultural knowledge, and schools’ endorsement of intercultural contact) as continuous variables in Mplus 8.3 (Muthén & Muthén, 1998–2019). LPA is a statistical person-centric approach that allows for the recovery of hidden groups from observed data (Oberski, 2016). The assumption is that “people can be *typed* with varying degrees of probabilities into categories (subpopulations) that have different configural profiles of personal and/or environmental attributes” (Spurk et al., 2020, pp. 1–2). Therefore, latent typologies are created based on data and the probability of everyone belonging to a specific subgroup. For each country, LPAs were conducted for the national overall sample first (see Appendix, Figs. 4–6). Then separate LPAs were calculated for the two subsamples in each country: students with and without a migration background. In deciding on the number of profiles in LPA, models with up to six latent profiles were examined, and model fit indices and theoretical considerations in the analysis of the various patterns guided the selection of the number of profiles in each sample (Geiser, 2009; Nylund et al., 2007). To assess the classification of participants, maximum likelihood estimation was applied with robust standard errors. In summary, three profiles were found in the three national samples and each of the national migration background and non-migration background subsamples: the mutual integration profile, with the highest agreement in all four dimensions (except for the adoption dimension in Switzerland); the mutual mild-integration profile, with the highest agreement with heritage culture maintenance; and the low-responsibility-on-majority profile, with the strongest disagreements with the two majority dimensions. The most common profile across countries and groups was the mutual integration profile and most students were found in one of the two integration profiles (see Table 3 for an overview).

**Table 3:** Overview findings acculturation profiles

Acculturation profile	Country	Migration background	Migration background students’ heritage culture maintenance	Migration background students’ dominant culture adoption	Majority students’ acquisition of cultural knowledge	Schools’ endorsement of intercultural contact
Mutual Integration	Germany	With (*n* = 113, 43%)	Agree	(Agree)	Agree	Agree
		Without (*n* = 45, 61%)	Agree	(Agree)	Agree	Agree
	Greece	With (*n* = 79, 40%)	Agree	Agree	Agree	Agree
		Without (*n* = 95, 42%)	Agree	Agree	Agree	Agree
	Switzerland	With (*n* = 105, 40%)	Agree	(Agree)	Agree	Agree
		Without (*n* = 40, 43%)	Agree	(Disagree)	Agree	Agree
Mutual Mild-Integration	Germany	With (*n* = 123, 47%)	Agree	(Disagree)	(Agree)	(Agree)
		Without (*n* = 24, 32%)	Agree	Disagree	(Disagree)	(Agree)
	Greece	With (*n* = 99, 51%)	Agree	(Agree)	(Agree)	Agree
		Without (*n* = 101, 44%)	Agree	(Agree)	(Agree)	Agree
	Switzerland	With (*n* = 145, 53%)	Agree	(Disagree)	(Agree)	(Agree)
		Without (*n* = 42, 46%)	Agree	(Agree)	(Agree)	(Agree)
Low-responsibility-on-majority	Germany	With (*n* = 26, 10%)	(Agree)	Disagree	Disagree	Disagree
		Without (*n* = 5, 7%)	Disagree	(Disagree)	Disagree	Disagree
	Greece	With (*n* = 18, 9%)	Disagree	(Disagree)	Disagree	Disagree
		Without (*n* = 32, 14%)	Agree	(Disagree)	Disagree	(Agree)
	Switzerland	With (*n* = 17, 7%)	Disagree	Disagree	Disagree	Disagree
		Without (*n* = 10, 12%)	(Agree)	(Agree)	Disagree	Disagree

Agree is defined as a value between 3 and 4; (Agree) relates to values between 2.5 and 2.9; (Disagree) relates to values between 2.1 and 2.4; and Disagree is defined as a value between 1 and 2

In Mplus, while running the LPAs, missing data were handled using full information maximum likelihood (FIML), except in the case of missing values on all variables (*n* = 10, 3% in Germany; *n* = 15, 3% in Greece; and *n* = 16, 4% in Switzerland). FIML uses all available data without imputing missing data, which may introduce randomness in the data. Thus, it is unbiased and preferable to other methods (Dong & Peng, 2013). Across the three countries and the two migration and non-migration background groups, missing data ranged from 0 to 7% in the first dimension on migration background students’ heritage culture maintenance (Germany *n* = 9, 4% migration background; *n* = 0, 0% non-migration background; Greece *n* = 13, 7% migration background; *n* = 5, 2% non-migration background; and Switzerland *n* = 3, 1% migration background; *n* = 0, 0% non-migration background); from 4 to 11% in the second dimension on migration background students’ dominant culture adoption (Germany *n* = 24, 10% migration background; *n* = 4, 5% non-migration background; Greece *n* = 19, 11% migration background; *n* = 10, 5% non-migration background; and Switzerland *n* = 11, 4% migration background; *n* = 5, 6% non-migration background); from 1 to 13% in the third dimension on non-migration background students’ cultural knowledge acquisition (Germany *n* = 14, 6% migration background; *n* = 1, 1% non-migration background; Greece *n* = 22, 13% migration background; *n* = 5, 2% non-migration background; and Switzerland *n* = 9, 4% migration background; *n* = 4, 5% non-migration background); and from 1 to 19% in the fourth dimension on schools’ endorsement of intercultural contact and exchange (Germany *n* = 15, 6% migration background; *n* = 1, 1% non-migration background; Greece *n* = 31, 19% migration background; *n* = 11, 5% non-migration background; and Switzerland *n* = 10, 4% migration background; *n* = 4, 5% non-migration background).

#### Germany

**Fig. 1:**
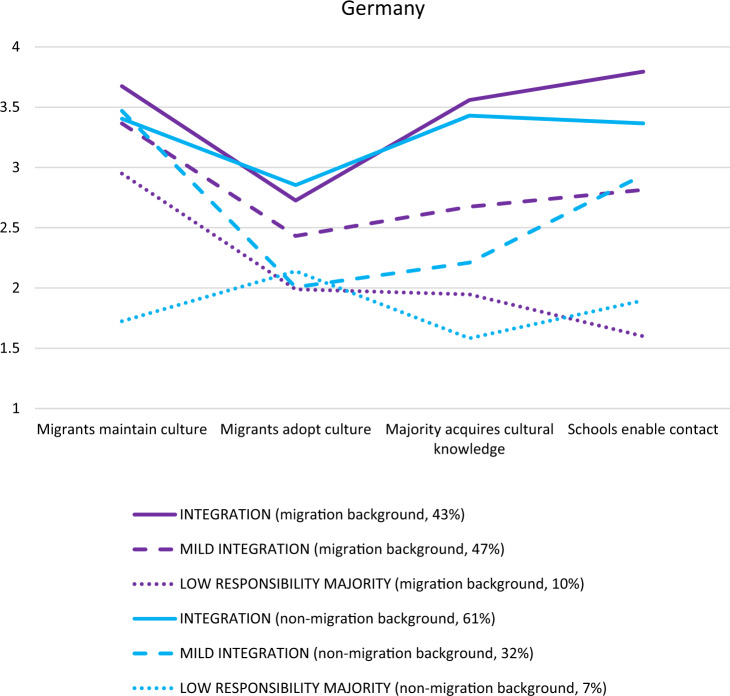
*Latent Profile Analysis of the German Subsamples With (n* = *262) vs. Without (n* = *74) Migration Background. Note*. Analyzing attitudes toward mutual acculturation via a four-dimensional framework provided three distinct profiles: mutual integration, mutual mild-integration, and low responsibility majority. 1 = *disagree*, 2 = *disagree somewhat*, 3 = *agree somewhat*, 4 = *agree*

Model fit (see Appendix, Table 5) was best for the three-profile solution in the whole sample (*N* = 336). In the two subsamples, for those with (*n* = 262) and without (*n* = 74) a migration background, although the three-profile solution did not significantly improve the model fit over the two-profile solution (see Appendix, Table 5), a three-profile solution was chosen because the third class presented a theoretically distinct profile, compared to a two-profile solution. In all three analyses, three similar profiles were found (see Fig. 1 and Appendix for the full sample figure). First, in the mutual integration profile (migration background subsample *n* = 113, 43%; non-migration background sample *n* = 45, 61%), students agreed or tended to agree with all four dimensions (agreement is shown as a value equal to or greater than 3 out of 4 on the *y*-axis; a tendency to agree is shown as a value equal to or greater than 2.5 on the *y*-axis). Moreover, the mutual integration profile was very similar for students with and without a migration background. Second, in the mutual mild-integration profile (migration background subsample *n* = 123, 47%; non-migration background sample *n* = 24, 32%), students agreed with most dimensions but less so than those in the mutual integration group. Moreover, the mutual mild-integration profile slightly differed across students with and without a migration background. Those without a migration background tended to disagree with immigrants’ need to adopt the dominant culture and the majorities’ need to acquire intercultural knowledge, compared to those with a migration background, who tended to agree with these dimensions. Most students in the German sample across both groups were in one of the integration profiles. Finally, the low-responsibility-on-majority profile consisted of a relatively smaller group of students (migration background subsample *n* = 26, 10%; non-migration background sample *n* = 5, 7%), who mostly disagreed with the two majority dimensions (i.e., that majority students should acquire cultural knowledge and schools should enable intercultural contact; disagreement is shown as a value equal to or lower than 2; a tendency to disagree is shown as a value lower than 2.5). This profile differed across groups mostly regarding the heritage culture maintenance dimension so that those without a migration background disagreed with migrants maintaining their culture, compared to those with a migration background (who agreed with this dimension). Across three profiles, students with a migration background varied the most in their attitudes toward schools’ endorsement of intercultural contact. Students without a migration background, however, varied the most in their attitudes on majority students’ acquisition of cultural knowledge.

#### Greece

**Fig. 2:**
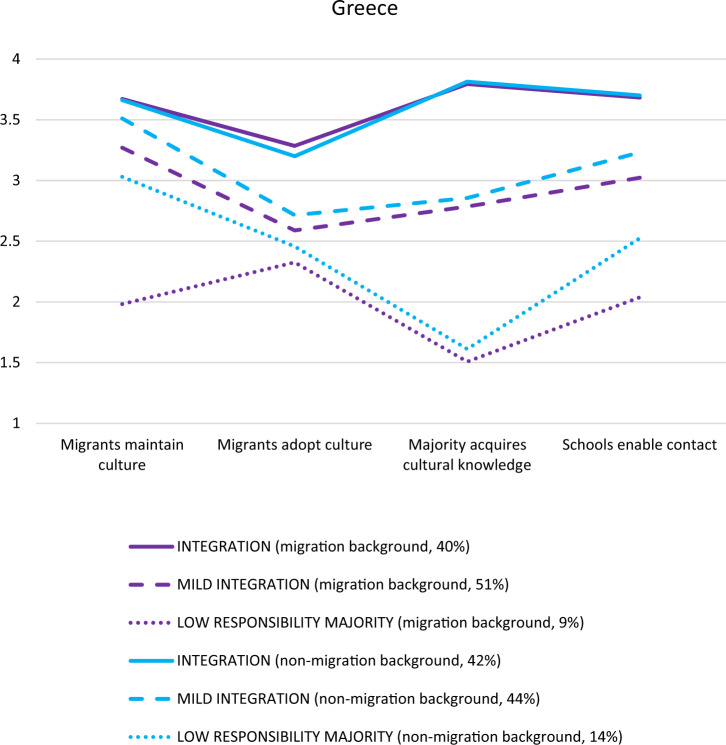
*Latent Profile Analysis of the Greek Subsamples With (n* = *196) vs. Without (n* = *228) Migration Background. Note*. Analyzing attitudes toward mutual acculturation via a four-dimensional framework provided three distinct profiles: mutual integration, mutual mild integration, and low responsibility majority. 1 = *disagree*, 2 = *disagree somewhat*, 3 = *agree somewhat*, 4 = *agree*

Model fit (see Appendix, Table 6) was best for the three-profile solution in the full sample (*N* = 424) and the subsample of students without a migration background (*n* = 228). In the subsample of those with a migration background (*n* = 196), although the three-class model did not significantly improve the model fit over a two-class model, similar to the subsample analyses in Germany, a three-profile solution was chosen because the third class also presented a theoretically distinct and meaningful profile. Model-fit indices were still good for the three-profile solution in this subsample. In all three analyses (full sample and two subsample analyses), three similar profiles were found (see Fig. 2, see Appendix for the full sample figure). First, among those in the mutual integration profile (migration background subsample *n* = 79, 40%; non-migration background sample *n* = 95, 42%), students agreed with all four dimensions, and those with and without a migration background did not differ from one another. Second, in the mutual mild-integration profile (migration background subsample *n* = 99, 51%; non-migration background sample *n* = 101, 44%), students agreed or tended to agree with all dimensions but most strongly so with the heritage culture maintenance dimension, that is, migrants are allowed to maintain their cultures. Overall, those in the mutual mild-integration profile agreed with the dimensions less strongly than those in the mutual integration profile. Moreover, those with and without a migration background did not differ from one another. As in the German sample, most students across both groups in the Greek sample were in either of the two integration profiles. Third, the low-responsibility-on-majority profile consisted of a smaller group of students (migration background subsample *n* = 18, 9%; non-migration background sample *n* = 32, 14%), who tended to disagree with most dimensions and strongly disagreed with the dimension concerning majority students’ acquisition of cultural knowledge. In three out of the four dimensions, students with and without a migration background did not differ, but in the dimension regarding migrants maintaining culture, surprisingly, those with a migration background disagreed, and those without a migration background agreed with it. Across the three profiles, students with and without a migration background varied the most in their attitudes toward majority students’ acquisition of cultural knowledge.

#### Switzerland

**Fig. 3:**
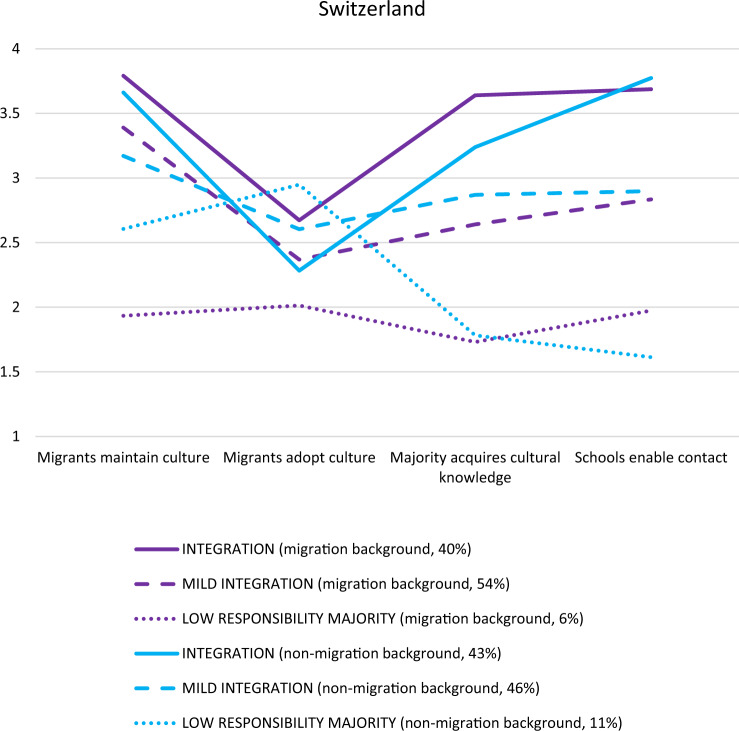
*Latent Profile Analysis of the Swiss Subsamples With (n* = *267) vs. Without (n* = *92) Migration Background. Note*. Analyzing attitudes toward mutual acculturation via a four-dimensional framework provided three distinct profiles: mutual integration, mutual mild integration, and low responsibility majority. 1 = *disagree*, 2 = *disagree somewhat*, 3 = *agree somewhat*, 4 = *agree*

**Fig. 4:**
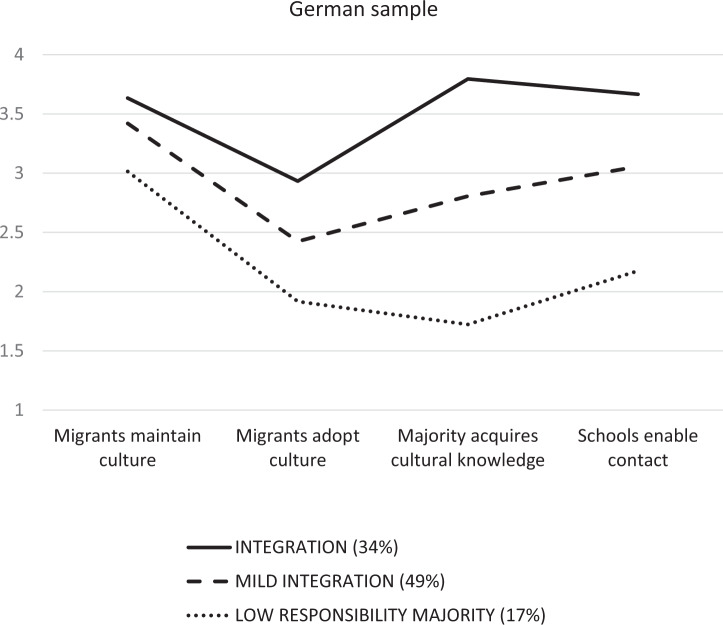
*Latent Profile Analysis of the German National Sample* (*n* = *336)*. Note: Analyzing attitudes toward mutual acculturation via a four-dimensional framework provided three distinct profiles: Integration, mild integration, and low responsibility majority. 1 = disagree, 2 = disagree somewhat, 3 = agree somewhat, 4 = agree

**Fig. 5:**
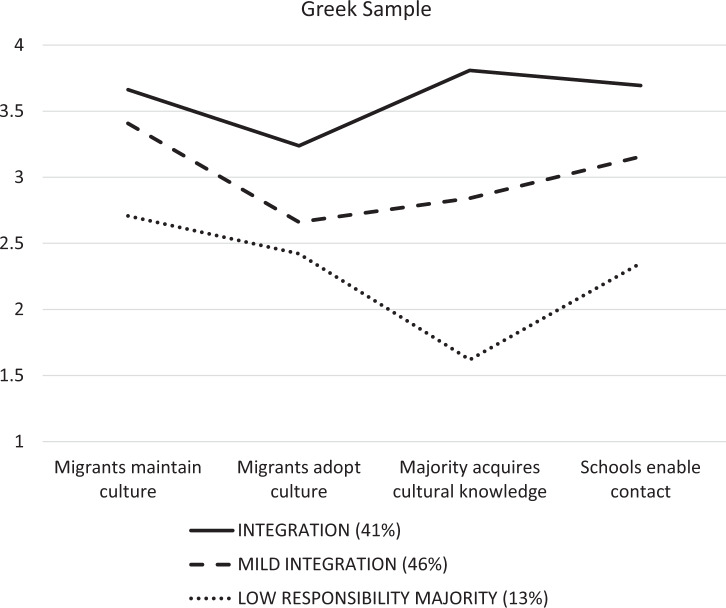
*Latent Profile Analysis of the Greek National Sample (*n = *424)*. Note: Analyzing attitudes toward mutual acculturation via a four-dimensional framework provided three distinct profiles: Integration, mild integration, and low responsibility majority. 1 = disagree, 2 = disagree somewhat, 3 = agree somewhat, 4 = agree

**Fig. 6:**
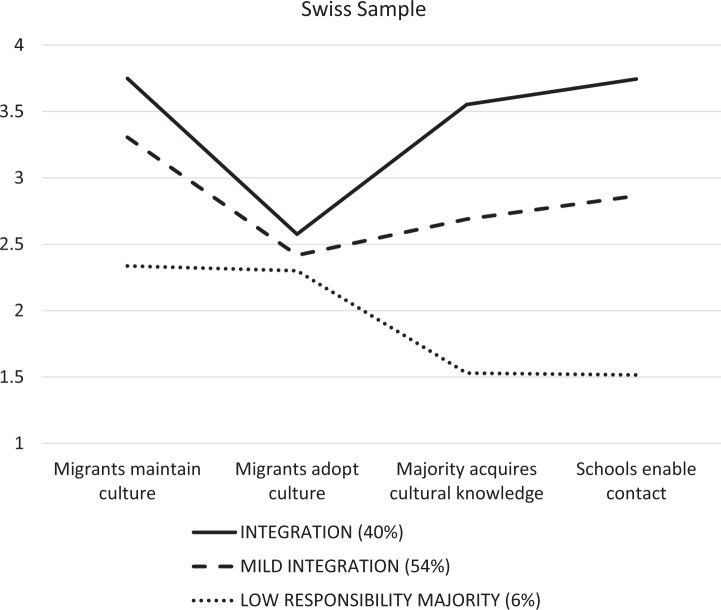
Latent Profile Analysis of the Swiss National Sample (n = 359). Note: Analyzing attitudes toward mutual acculturation via a four-dimensional framework provided three distinct profiles: Integration, mild integration, and low responsibility majority. 1 = disagree, 2 = disagree somewhat, 3 = agree somewhat, 4 = agree

The three-profile model was again opted for in the full sample (*N* = 359; see Appendix, Table 7). Comparing the three-profile with the two-profile solution, the three-profile solution improved the model fit and the three profiles were theoretically distinct and meaningful. Although the four-profile model still provided a significant improvement over the three-profile model in terms of model fit, the fourth profile did not add a theoretically distinct group. In the two subsamples, those with (*n* = 267) and without (*n* = 92) a migration background, a three-profile model was chosen. Among those with a migration background, a three-profile model provided the best fit both in terms of model fit indices and theoretically. Among those without a migration background, although model fit indices were similar for two- and three-class solutions, the third class still added a meaningful and distinct group. In all three samples, three similar profiles were found (see Fig. 3, see Appendix for the full sample figure). First, in the mutual integration profile (migration background subsample *n* = 105, 40%; non-migration background sample *n* = 40, 43%), students largely agreed with the four dimensions but much less so with the dimension concerning migrants adopting culture. Although students with and without a migration background were mostly similar in their attitudes, particularly in the adoption dimension, responses of students with a migration background were slightly above the scale’s midpoint (on the agree side) whereas students without a migration background were slightly below the scale’s midpoint (on the disagree side). Second, in the mutual mild-integration profile (migration background subsample *n* = 145, 53%; non-migration background sample *n* = 42, 46%), students largely agreed with all dimensions but more strongly so with the heritage culture maintenance dimension. Overall, those in the mutual mild-integration profile agreed with the dimensions less strongly than those in the mutual integration profile. Moreover, students with and without a migration background were largely similar in their attitudes. Similar to the other countries, most students in the Swiss sample across both groups fit one of the integration profiles. Third, the low-responsibility-on-majority profile consisted of a smaller group of students (migration background subsample *n* = 17, 7%; non-migration background sample *n* = 10, 12%), who disagreed with the two majority dimensions (majority students’ acquisition of cultural knowledge and schools’ endorsement of intercultural contact). However, students with and without a migration background differed in their expectations of migrants: Although those without a migration background agreed with both migrant dimensions, more so with migrants adopting the culture, those with a migration background tended to disagree with these dimensions. Additionally, across three profiles, students with and without a migration background varied the most in their attitudes concerning both majority dimensions.

### Acculturation Profiles and Psychological Adjustment

To answer the second research question, several univariate ANOVAs were run in SPSS (version 27) to determine whether students with and without a migration background in the three profiles of mutual acculturation (integration, mild integration, and low-responsibility majority) significantly differed in terms of their psychological adjustment (see Table 4). In each country, to allow for the group-specific patterns of students with and without a migration background, the latent profile solution of each subgroup was fused into one. Therefore, the variable used for the ANOVA comprises the three profiles, each of which consists of the respective profile of students with and without a migration background. The effects of the found acculturation profiles of students with and without a migration background on self-esteem and self-determination were assessed while controlling for gender. The interactions of acculturation profiles with a migration background were tested. When comparing mean differences of the three acculturation profiles (post hoc tests), Bonferroni correction and bootstrap of 1000 samples were used. Bootstrap confidence intervals for the mean differences are reported in addition to *p* values. In summary, the acculturation profiles differed significantly concerning psychological adjustment in Germany and Switzerland but not in Greece. Those with and without a migration background significantly differed in psychological adjustment in Germany and Greece but not in Switzerland. The interaction between the two was significant in self-esteem and self-determination in Germany. However, neither country nor having a migration background changed the finding that those in the integration profile had higher psychological adjustment than those in the mild-integration and/or those in the low-responsibility-on-majority groups, and those in the mild-integration profile had higher psychological adjustment than those in the low-responsibility-on-majority group although the differences between the latter two were not always significant.

**Table 4:** ANOVA summary table for psychological adjustment

	DV	Self-Esteem			Self-Determination		
Country	IV	*F* (df, e)	*p*	Effect Size	*F* (df, e)	*p*	Effect Size
Germany	Acculturation attitudes LPA	6.29 (2, 316)	0.002	0.038	4.80 (2, 319)	0.009	0.029
	Migration background	7.50 (1, 316)	0.007	0.023	2.71 (1, 319)	0.101	0.008
	LPA × Migration	4.69 (2, 316)	0.010	0.029	3.73 (2, 319)	0.025	0.023
	R^2^			0.09			0.05
Greece	Acculturation attitudes LPA	0.69 (2, 349)	0.505	0.004	0.25 (2, 390)	0.781	0.001
	Migration background	6.36 (1, 349)	0.012	0.018	10.15 (1, 390)	0.002	0.025
	LPA × Migration	0.79 (2, 349)	0.453	0.005	2.92 (2, 390)	0.055	0.015
	R^2^			0.03			0.04
Switzerland	Acculturation attitudes LPA	3.48 (2, 337)	0.032	0.020	7.10 (2, 347)	<0.001	0.039
	Migration background	0.01 (1, 337)	0.921	0.000	0.65 (1, 347)	0.422	0.002
	LPA × Migration	0.30 (2, 337)	0.743	0.002	2.98 (2, 347)	0.052	0.017
	R^2^			0.05			0.06

In SPSS, when running the ANOVAs, listwise deletion was used as the default option. Due to the missingness in control variables and outcome variables, the missing values ranged from 6 to 19% across different sets of analyses (*n* = 23, 7% for self-esteem and *n* = 20, 6% for self-determination in Germany; *n* = 83, 19% for self-esteem and *n* = 42, 10% for self-determination in Greece; and *n* = 31, 8% for self-esteem and *n* = 21, 6% for self-determination in Switzerland).

In a first analysis, it was also tested whether the acculturation profiles differed in terms of the teacher support they experienced. Similar to psychological adjustment, significant results were found only in Germany and Switzerland: those in the integration profile reported higher teacher support than those in the mild-integration profile and those in the low-responsibility-on-majority profile in Germany, and those in the integration profile reported higher teacher support than those in the low-responsibility-on-majority profile in Switzerland. Yet, to focus the findings on psychological adjustment, these results were excluded from this publication.

Sensitivity analyses were run in G*Power (3.1.9.7) and found that ANOVAs with *n* = 323–397 participants across six groups with one covariate would be sensitive to effects with an effect size of 0.22 in Germany, 0.20–0.21 in Greece, and 0.21 in Switzerland with 80% power (alpha = 0.05). This means that the study was not able to reliably detect effects with an effect size smaller than 0.20–0.22 across the three countries.

#### Germany

Acculturation profiles significantly differed in terms of self-esteem and self-determination. Students with and without a migration background significantly differed in self-esteem. Finally, the interaction of migration background and acculturation profiles showed a significant effect on self-esteem and self-determination.

Looking at acculturation profiles more specifically, those in the integration profile (*M* = 2.95, *SD* = 0.55, *p* = 0.004, 95% CI [0.15, 0.73]) and those in the mild-integration profile (*M* = 2.91, *SD* = 0.55, *p* = 0.014, 95% CI [0.02, 0.62]) reported significantly higher self-esteem than those in the low-responsibility-on-majority profile (*M* = 2.74, *SD* = 0.54). Moreover, those in the integration profile (*M* = 2.87, *SD* = 0.41) reported higher self-determination than those in the mild-integration (*M* = 2.77, *SD* = 0.41, *p* = 0.024, 95% CI [0.02, 0.22]) and low-responsibility-on-majority profiles (*M* = 2.76, *SD* = 0.43, *p* = 0.006, 95% CI [0.12, 0.44]), but those in the mild-integration profile reported significantly higher self-determination than those in the low-responsibility-on-majority profile (*p* = 0.047, 95% CI [−0.02, 0.34]). Overall, those in the integration profile reported higher psychological adjustment than the two other groups.

Looking at students with and without a migration background, those with a migration background (*M* = 2.93, *SD* = 0.53) had significantly higher self-esteem than those without a migration background (*M* = 2.85, *SD* = 0.59).

These main effects were qualified by the significant interactions between acculturation profiles and migration background, which showed that the significant differences in self-esteem and self-determination only emerged for the non-migration background subsample. Those in the integration profile (*M* = 3.00, *SD* = 0.52, *p* = 0.001, 95% CI [0.35, 1.34]) and those in the mild-integration profile (*M* = 2.73, *SD* = 0.61, *p* = 0.004, 95% CI [0.02, 1.17]) reported higher self-esteem than those in the low-responsibility group (*M* = 2.06, *SD* = 0.45). Students without a migration background in the integration profile (*M* = 2.94, *SD* = 0.32) reported higher self-determination than those in the mild-integration group (*M* = 2.75, *SD* = 0.40, *p* = 0.051, 95% CI [0.01, 0.35]) and those in the low-responsibility group (*M* = 2.34, *SD* = 0.23, *p* = 0.001, 95% CI [0.34, 0.81]). Those in the mild-integration group (*M* = 2.75, *SD* = 0.40) reported higher self-determination than those in the low-responsibility group (*M* = 2.34, *SD* = 0.23, *p* = 0.002, 95% CI [0.11, 0.67]). Although significant, the mean differences, particularly in self-determination, were overall small.

#### Greece

Acculturation profiles did not significantly differ in terms of self-esteem and self-determination. Students with and without a migration background significantly differed in self-esteem and self-determination. No significant interaction effects were found.

Looking at the effects of migration background, those without a migration background reported higher self-esteem (*M* = 3.06, *SD* = 0.50) and higher self-determination (*M* = 2.89, *SD* = 0.43) than those with a migration background (*M* = 2.93, *SD* = 0.51 for self-esteem; *M* = 2.77, *SD* = 0.40 for self-determination). Although significant, the mean differences were again overall small.

#### Switzerland

Although acculturation profiles significantly differed in terms of self-esteem and self-determination, students with and without a migration background did not significantly differ, and no interaction effects were found.

Looking at acculturation profiles, those in the integration profile (*M* = 3.06, *SD* = 0.57) reported significantly higher self-esteem than those in the mild-integration (*M* = 2.88, *SD* = 0.52, *p* = 0.017, 95% CI [0.03, 0.28]) and low-responsibility-on-majority profiles (*M* = 2.82, *SD* = 0.44, *p* = 0.014, 95% CI [0.05, 0.44]). Similarly, those in the integration profile (*M* = 3.01, *SD* = 0.42) reported higher self-determination than those in the mild-integration (*M* = 2.83, *SD* = 0.44, *p* = 0.003, 95% CI [0.05, 0.25]) and low-responsibility-on-majority profiles (*M* = 2.74, *SD* = 0.52, *p* = 0.002, 95% CI [0.11, 0.56]).

## Discussion

Acculturation is often misconceived as a process that only minority members experience. As a result, little is known as to how majority members acculturate (but see Kunst et al., 2021). The present study goes beyond assessing either minority or majority acculturation by combining them into a four-dimensional assessment of attitudes toward both minority and majority acculturation within the school context. Thus, both minority and majority adolescents’ attitudes toward *migration background students’ heritage culture maintenance* and *dominant culture adoption*, as well as toward *majority students’ acquisition of cultural knowledge* and *schools’ endorsement of intercultural contact* (Sidler et al., 2021) were assessed. First, through a multigroup CFA, measurement invariance of the four-dimensional assessment of attitudes toward mutual acculturation was established across Germany, Greece, and Switzerland (see Supplementary Material for results). Thus, comparisons across the three samples using this measure proved meaningful and valid. The main aims of this study were twofold: first, to analyze adolescents’ attitudes toward mutual acculturation in these three European countries, and second, to assess the association between these acculturation profiles and psychological adjustment. Each aim will be discussed in the next sections.

### Mutual Acculturation for Adolescents with and without a Migration Background

In relation to the first research question, the study sought to assess how and to what extent adolescents’ attitudes toward mutual acculturation differ within and across minority and majority groups. Three acculturation profiles were found in each country and each group: the mutual integration profile, with the highest agreement in all four dimensions; the mutual mild-integration profile, with a high agreement with heritage culture maintenance and fluctuating agreement to the other three dimensions relative to context and group; and the low-responsibility-on-majority profile, with the strongest disagreements with the two majority dimensions (see Table 3). The most common profile across countries and groups was the mutual integration profile and most students were found in one of the two integration profiles. The third profile low-responsibility-on-majority showed most cross-national variations, which reflect the three different national contexts (e.g., MIPEX). These results add a more nuanced understanding of acculturation to those of previous studies focusing only on attitudes toward minority acculturation, as the acculturation profiles in this study were based on the assessment of attitudes toward mutual acculturation. By assessing attitudes toward mutual acculturation, this study shows how integration profiles are more diverse than would be expected by traditional assessments of bicultural attitudes that focus only on attitudes toward minority acculturation. These findings strengthen previous research using LPA in acculturation research as a robust person-centric technique to model acculturation without anticipating profiles beforehand (Fox et al., 2013). Moreover, the three profiles differed most concerning the two majority dimensions, which stresses the importance of a mutual acculturation framework, as the two new dimensions are distinct features of the three profiles.

Adolescence is a time when opinions and attitudes are formed and developed, rendering research on attitudes toward mutual acculturation key to understanding acculturation expectations. One common finding across contexts and groups in this study was that most adolescents fit one of the two integration profiles, namely in Germany (90% of those with and 94% of those without a migration background), Greece (91% of those with and 86% of those without a migration background), and Switzerland (93% of those with and 89% of those without a migration background). This aligns with previous research on migration background adolescents finding that they favor integration (Makarova & Birman, 2015, 2016). Concerning non-migration background majority adolescents, this openness could stem from their young age. Given the rise of migration in the 21st century, they may have experienced other cultures at an earlier age and therefore have a more open-minded mindset. This might also be the case for migration background adolescents, as in a study in Sweden, adolescents were found to become more open to diverse perspectives over time (Bayram Özdemir et al., 2021), stressing a developmental perspective during adolescence. Another commonality across the two integration profiles is that all students in an integration profile agreed with schools’ endorsement of intercultural contact, even though a weaker agreement is found in the mild-integration profiles. The latter strengthens findings concerning schools being a shared ground for promoting openness to diversity (Bayram Özdemir et al., 2021) and therefore for schools to actively shape intercultural contact and exchange. Similar to the schools’ endorsement of intercultural contact dimension, there is a strong agreement across the integration profiles in relation to migration background students’ maintenance of heritage culture. In particular, adolescents with a migration background across all three countries agree with the heritage culture maintenance dimension, thus demonstrating the importance of making space for heritage culture maintenance within the context of school. Yet, there were also slight cross-national differences: Across all three profiles, it is only the majority students in Greece and Switzerland and the migration background students in Germany who agree with the first dimension concerning heritage culture maintenance no matter which pattern they fit. This finding is particularly interesting in the Swiss context, as Switzerland’s adult majority has voted to ban burqas and minarets in the last decade (Arlt, 2021; Dodd, 2015). In Germany, the agreement of all students with a migration background with the first dimension on heritage culture maintenance probably reflects their experience on heritage culture maintenance. This result aligns with previous findings on minority adolescents in Germany stressing their culture maintenance (Dimitrova et al., 2015). Most cross-country variation across the two integration profiles was found in relation to migration background students’ adoption of the local dominant culture and majority students’ acquisition of cultural knowledge, which could relate to the diverse national contexts following the MIPEX, the unfamiliarity with the concept of majority acculturation, and the emphasis on context in acculturation research (Birman & Simon, 2014).

The differences across groups and countries emerged particularly in the low-responsibility-on-majority profile, shifting between separation (agreement only with heritage culture maintenance), individualism (disagreement with all four dimensions), and one-sided integration (agreement with the two minority acculturation dimensions: heritage culture maintenance and dominant culture adoption). However, given the small number of adolescents in each pattern, these differences should be analyzed with caution. Among those endorsing this profile, students with a migration background show an individualism pattern in Greece and Switzerland and a separation pattern in Germany. Students without a migration background show an individualism pattern in Germany, a one-sided integration pattern in Switzerland (disagreeing with the two majority dimensions), and a separation/integration pattern in Greece (fully disagreeing only with majority students acquiring cultural knowledge). An individualism pattern could be due to adolescents focusing on individualism or color-blindness (Bourhis et al., 1997; Dovidio et al., 2016) or, due to globalization and digitalization, such that they prefer a global mindset instead of the focus on divisions into ethnocultural groups. This kind of global mindset could also explain why this pattern was found for both groups of students—those with and without a migration background. Yet, migration background students’ disagreement with heritage culture maintenance (i.e., having an individualism pattern like in Greece and Switzerland) could also be due to their experience of assimilationist pressures within the school context (Archakis et al., 2018; Makarova et al., 2019) or unfavorable integration policies (e.g., MIPEX). Then, the one-sided integration profile of majority students strengthens the importance of a mutual acculturation framework, because if only attitudes toward minority acculturation had been assessed, these students would have fallen into an integration category. Yet, in comparison to the majority students in one of the integration profiles, the majority students in the one-sided integration profile are a distinct group in relation to how they expect the majority to acculturate. Given that integration policies do not focus on majority members, this disagreement with majority students’ acquisition of cultural knowledge could stem from the unfamiliarity with this concept. The common factor in the low-responsibility-on-majority profiles lies in the small group sizes across countries and groups and the strong disagreement with the two majority dimensions (except for majority students in Greece only disagreeing strongly with the third dimension).

### Mutual Acculturation and Psychological Adjustment

Concerning the second research question, the associations between the aforementioned acculturation profiles and psychological adjustment were assessed. Psychological adjustment was measured via self-esteem and self-determination. An integration or bicultural orientation was predominantly positively associated with adjustment for students with a migration background (Makarova & Birman, 2015, 2016), and very few results have been established for students without a migration background (Haugen & Kunst, 2017). Yet, the results for students with a migration background were also inconsistent, which could stem from contextual factors influencing acculturation (Birman & Simon, 2014). The findings of this study strengthen the integration hypothesis that having a mutual integration strategy is associated with positive psychological adjustment. Namely, in Switzerland, those in the mutual integration group reported higher self-esteem and higher self-determination than those in the other two groups, and no significant interaction effects with migration background were found. This relates to and enhances previous research in Switzerland finding a positive relationship between psychosocial adaptation and integration orientations for minority adolescents (Hoti et al., 2017). Yet, the results also indicate important contextual variety: In Germany, integration and mild-integration groups reported higher self-esteem than the low responsibility group; strong- and mild-integration groups reported higher self-determination than the mild-integration and low-responsibility-on-majority groups. However, through interaction effects of migration background and acculturation profiles, the significant differences in self-esteem and self-determination only emerged for the non-migration-background subsample. Thus, in Germany, the benefits of a mutual integration profile were more obvious for students without a migration background. A study in Norway found that majority members’ openness predicted more adoption of minority cultures (Kunst et al., 2021), thus high self-esteem and self-determination of German majority students might relate to mutual integration attitudes and not vice versa. Future longitudinal research in the German context would be needed to gain more clarity on the directionality between attitudes toward mutual acculturation and positive psychological adjustment. In Greece, however, psychological adjustment in terms of self-esteem and self-determination was not significantly associated with the acculturation profiles regardless of students’ migration background. This could relate to previous findings on contextual variability in the adaptive value of integration (Phalet & Baysu, 2020), meaning that in less welcoming contexts, an integration strategy might not prove to be beneficial. It could also mean that the relationship between acculturation attitudes and adjustment is not as strong as supposed (Bierwiaczonek & Kunst, 2021). Yet, an ANOVA with *n* = 356 (self-esteem) respectively *n* = 397 (self-determination) participants across six groups with one covariate would be sensitive to effects with an effect size of 0.21 and 0.20 respectively with 80% power (alpha = 0.05). This means that the study was not able to reliably detect effects with an effect size smaller than 0.21 and 0.20 respectively. Thus, there might be a small relation between acculturation profiles and psychological adjustment in Greece, yet this study might not have detected it.

The findings of this study go beyond previous research, as having a strong mutual integration strategy relates to agreeing strongly not only with minority acculturation but also with majority acculturation. Additionally, whereas previous research focused on minority adolescents’ adjustment, the results of this study indicate a relationship between a mutual integration orientation and positive psychological adjustment not only for adolescents with a migration background but also or even only for adolescents without a migration background. Additionally, schools are expected to enhance intercultural contact in the two mutual integration profiles, which aligns with students having higher self-esteem, well-being, and motivation to learn in schools where diversity is valued and discussed (Vedder & van Geel, 2012). The cross-national diversity demonstrates that researchers should consider perspectives and context (Birman & Simon, 2014) when assessing acculturation attitudes and psychological adjustment. Moreover, longitudinal research designs are needed to understand the relationship and directionality between acculturation attitudes and positive adjustment better (Bierwiaczonek & Kunst, 2021).

### Strengths, Future Directions, and Limitations

Through a four-dimensional exploration of acculturation attitudes combining minority and majority acculturation, the findings of this study enhance acculturation-attitude theory with latent patterns assessed using a mutual acculturation framework based on minority and majority acculturation. Moreover, this study’s results empirically substantiate the importance of combining minority and majority acculturation, as both are important for students with and without a migration background. Hence, this study shows the inherent mutuality of acculturation and the shared responsibility of minority and majority members, including institutions, to enhance successful acculturation. Therefore, further studies assessing acculturation attitudes and psychological adjustment should also include majority acculturation, as this study’s results emphasize the importance of the four dimensions for students with and without a migration background.

Practical implications arise in the context of school. Although diverse associations between attitudes toward mutual acculturation and psychological adjustment were found, the overall patterns are very similar across groups and countries. Namely, a strong relationship emerged between positive psychological adjustment and mutual integration, meaning the expectations placed on minority and majority adolescents as well as on schools to integrate. Most of the participating students saw schools as actors within the process of acculturation and found it important that schools enable intercultural contact, emphasizing schools and teachers’ ability to enable intergroup contact and interactions (Phalet & Baysu, 2020) while promoting minority and majority students learning about each other’s cultures.

However, two limitations should be addressed: First, given the study’s cross-sectional design, neither directional nor causal statements can be made about the relationship between attitudes toward mutual acculturation and psychological adjustment, which researchers should study using a longitudinal design (Bierwiaczonek & Kunst, 2021). Second, the categorization based on migration background allows for differentiation between students who belong to the dominant national majority and those who do not, accounting for power relations and practices aiming at legitimizing the majority’s dominant societal position (Connell, 1998). However, such categorization leads to loss of information, as migrants can be diverse regarding migration generation, migrant status, and countries of origin (Nauck & Genoni, 2019). Additionally, it may be problematic, as self-identification is not taken into account, and students might count as having a migration background but say otherwise (Horvath, 2019). Nevertheless, as this four-dimensional assessment of attitudes toward mutual acculturation relates to the acculturation of minority and majority students, assessing the perspective of minority and majority students (without dissecting the minority group further) promises insights into whether and how societal power relations affect the assessed attitudes toward mutual acculturation.

## Conclusion

Acculturation is a mutual process, rendering it important to study acculturation attitudes not only from minority and majority perspectives but also toward minority and majority acculturation. This study explored adolescents’ attitudes toward mutual acculturation and assessed the relationship between these acculturation profiles and psychological adjustment. Three distinct acculturation profiles were found for students with and without a migration background in Germany, Greece, and Switzerland, namely, two mutual integration profiles (strong vs mild) in which both immigrant background and majority students are expected to integrate, and a third one assuming lower responsibility for the majority. The patterns looked very similar across groups and countries and most adolescents fit in with one of the mutual integration profiles, suggesting similar developmental processes across contexts. Across the three profiles, students’ opinions varied most among the majority dimensions, that is, whether majority adolescents should acquire intercultural knowledge and whether schools should endorse intercultural contact. Then, the most notable differences were found in the low-responsibility-on-majority profiles, ranging from separation, individualism, and one-sided integration patterns across countries and groups. Finally, acculturation profiles mattered in terms of adolescents’ psychological adjustment, although this varied across groups and countries. In Germany and Switzerland, those in the strong integration groups reported the highest and those in the low-responsibility-on-majority group the lowest psychological adjustment, whereas those in the mild-integration group were somewhere in between. In Greece, however, integration profiles did not significantly benefit adolescents’ psychological adjustment. Overall, our findings stress the value of a mutual acculturation framework, which includes not only the majority and minority perspective but also minority and majority acculturation. Practically, these findings stress that most adolescents with and without a migration background are in favor of mutual integration and expect schools to enhance intercultural contact and exchange at school.

### Supplementary information

Supplementary Materials

## Appendix

Latent Profile Analyses with National Samples

Figures 4–6

Model Fit Indices for Latent Profile Analyses

Tables 5–7

**Table 5:** Model fit indices for latent profile analyses of the German national, migration background, and non-migration background samples

	#	Log likelihood	AIC	BIC	aBIC	Entropy	aLMR *p* value	BLRT *p* value	Sample proportion per class	Classification accuracy
GER nat	1	−1454.697	2925.393	2955.930	2930.553				336	
	2	−1362.122	2750.245	2799.867	2758.629	0.732	<0.001	<0.001	93 (29%); 243 (71%)	0.882–0.938
	**3**	−**1320.774**	**2677.548**	**2746.256**	**2689.158**	**0.803**	**<0.01**	**<0.001**	**165 (49%); 58 (17%); 113 (34%)**	0.894–0.940
	4	−1289.928	2625.856	2713.650	2640.691	0.837	0.22	<0.001	19 (6%); 43 (14%); 114 (34%); 160 (46%)	0.883–0.934
	5	−1261.933	2579.866	2686.745	2597.926	0.892	<0.05	<0.001	63 (19%); 13 (4%); 139 (40%); 108 (33%); 13 (4%)	0.904–0.962
	6	−1246.084	2558.167	2684.132	2579.452	0.895	0.34	<0.001	12 (4%); 129 (38%); 12 (4%); 104 (31%); 67 (20%); 12 (3%)	0.790–0.978
GER mbg	1	−1115.923	2247.845	2276.392	2251.029				262	
	2	−1037.841	2101.681	2148.070	2106.854	0.734	<0.01	<0.001	76 (31%); 186 (69%)	0.897–0.932
	**3**	−**1001.596**	**2039.192**	**2103.422**	**2046.354**	**0.844**	**0.15**	**<0.001**	**113 (43%); 26 (10%); 123 (47%)**	**0.895**–**0.957**
	4	−972.185	1990.369	2072.441	1999.521	0.879	<0.05	<0.001	35 (15%); 110 (41%); 14 (6%); 103 (39%)	0.910–0.956
	5	−951.417	1958.834	2058.747	1969.975	0.880	0.30	<0.001	94 (36%); 7 (3%); 22 (8%); 110 (41%); 29 (12%)	0.858–0.996
	6	−934.295	1934.590	2052.345	1947.720	0.890	0.11	<0.001	20 (7%); 86 (33%); 3 (1%); 108 (41%); 10 (4%); 35 (14%)	0.870–0.999
GER non-mbg	1	−336.477	688.953	707.386	682.175				74	
	2	−317.607	661.213	691.166	650.198	0.961	<0.05	<0.001	68 (92%); 6 (8%)	0.975–0.994
	**3**	−**306.743**	**649.485**	**690.958**	**634.233**	**0.799**	**0.38**	**<0.001**	**24 (32%); 5 (7%); 45 (61%)**	**0.873–0.982**
	4	−298.848	643.697	696.690	624.209	0.862	0.45	0.14	5 (7%); 31 (43%); 26 (36%); 12 (15%)	0.871–0.995
	5	−288.991	633.982	698.496	610.257	0.906	0.32	<0.05	5 (7%); 7 (10%); 33 (43%); 4 (6%); 25 (35%)	0.919–0.996
	6	−281.914	629.828	705.862	601.866	0.924	<0.05	0.17	4 (5%); 1 (2%); 7 (10%); 33 (43%); 4 (6%); 25 (35%)	0.932–1.000

Classification accuracy relates to the average latent class probabilities. Model fit for the chosen profile solution is in bold

*GER* Germany, *nat* national sample, *mbg* migration background sample, *non-mbg* non-migration background sample, *AIC* Akaike information criterion, *BIC* Bayesian information criterion, *aBIC* Sample-size adjusted BIC, *aLMR LRT* Lo-Mendell-Rubin adjusted likelihood ratio test, *BLRT* bootstrap likelihood ratio test

**Table 6:** Model fit indices for latent profile analyses of the Greek national, migration background, and non-migration background samples

	#	Log likelihood	AIC	BIC	aBIC	Entropy	aLMR *p*-value	BLRT *p*-value	Sample proportion per class	Classification accuracy
GRE nat	1	−1745.532	3507.063	3539.461	3514.074				424	
	2	−1621.568	3269.136	3321.782	3280.529	0.868	<0.05	<0.001	70 (18%); 354 (83%)	0.921–0.973
	**3**	−**1558.662**	**3153.323**	**3226.218**	**3169.098**	**0.851**	**<0.001**	**<0.001**	**56 (13%); 196 (46%); 172 (41%)**	0.928–0.942
	4	−1525.927	3097.854	3190.998	3118.011	0.844	0.29	<0.001	33 (8%); 22 (6%); 207 (48%); 162 (38%)	0.863–0.929
	5	−1494.859	3045.719	3159.111	3070.257	0.820	0.23	<0.001	35 (8%); 165 (39%); 171 (38%); 37 (11%); 16 (5%)	0.858–0.954
	6	−1464.835	2995.670	3129.311	3024.590	0.833	0.58	<0.001	24 (6%); 38 (11%); 161 (36%); 32 (7%); 159 (37%); 10 (3%)	0.805–0.900
GRE mbg	1	−793.946	1603.892	1630.117	1604.774				196	
	2	−727.751	1481.503	1524.118	1482.936	0.863	<0.001	<0.001	151 (77%); 45 (23%)	0.932–0.976
	**3**	−**692.776**	**1421.552**	**1480.558**	**1423.535**	**0.831**	**0.27**	**<0.001**	**18 (9%); 99 (51%); 79 (40%)**	**0.875**–**0.932**
	4	−671.921	1389.841	1465.238	1392.376	0.867	0.23	<0.001	77 (38%); 78 (40%); 31 (16%); 10 (5%)	0.875–0.941
	5	−659.430	1374.861	1466.648	1377.947	0.881	0.31	<0.001	8 (5%); 78 (40%); 32 (16%); 76 (38%); 2 (1%)	0.818–0.948
	6	−647.190	1360.379	1468.557	1364.016	0.860	0.39	<0.05	39 (19%); 2 (1%); 67 (34%); 9 (5%); 3 (2%); 76 (38%)	0.832–0.993
GRE non-mbg	1	−936.238	1888.475	1915.910	1890.555				228	
	2	−872.993	1771.987	1816.568	1775.367	0.813	<0.05	<0.001	39 (19%); 189 (81%)	0.906–0.955
	**3**	−**841.517**	**1719.034**	**1780.762**	**1723.714**	**0.877**	**<0.05**	**<0.001**	**32 (14%); 95 (42%); 101 (44%)**	**0.941–0.963**
	4	−815.422	1676.843	1755.718	1682.824	0.883	0.38	<0.001	17 (7%); 120 (51%); 81 (37%); 10 (4%)	0.909–0.947
	5	−791.641	1639.282	1735.304	1646.563	0.911	0.22	<0.001	94 (42%); 14 (6%); 19 (8%); 12 (5%); 89 (39%)	0.903–0.952
	6	−774.687	1615.374	1728.542	1623.954	0.865	0.50	<0.001	10 (5%); 13 (5%); 8 (3%); 89 (38%); 85 (38%); 23 (11%)	0.870–0.950

Classification accuracy relates to the average latent class probabilities. Model fit for the chosen profile solution is in bold

*GRE* Greece, *nat* national sample, *mbg* migration background sample, *non-mbg* non-migration background sample, *AIC* Akaike information criterion, *BIC* Bayesian information criterion, *aBIC* Sample-size adjusted BIC, *aLMR LRT* Lo-Mendell-Rubin adjusted likelihood ratio test, *BLRT* Bootstrap likelihood ratio test

**Table 7:** Model fit indices for latent profile analyses of the Swiss national, migration background, and non-migration background samples

	#	Log likelihood	AIC	BIC	aBIC	Entropy	aLMR *p*-value	BLRT *p*-value	Sample proportion per class	Classification accuracy
SWI nat	1	−1550.784	3117.567	3148.634	3123.254				359	
	2	−1444.806	2915.611	2966.094	2924.852	0.867	<0.05	<0.001	51 (15%); 308 (85%)	0.890–0.975
	**3**	−**1394.488**	**2824.976**	**2894.876**	**2837.771**	**0.778**	**0.06**	**<0.001**	**22 (6%); 195 (54%); 142 (40%)**	0.895–0.956
	4	−1351.172	2748.344	2837.660	2764.693	0.905	<0.01	<0.001	173 (47%); 14 (4%); 132 (37%); 40 (12%)	0.931–0.997
	5	−1326.829	2709.657	2818.390	2729.560	0.872	<0.01	<0.001	39 (12%); 15 (4%); 80 (23%); 175 (47%); 50 (15%)	0.893–0.975
	6	−1301.328	2668.656	2796.806	2692.113	0.861	0.21	<0.001	11 (3%); 161 (44%); 33 (10%); 25 (7%); 79 (21%); 50 (15%)	0.869–0.946
SWI mbg	1	−1168.322	2352.645	2381.343	2355.978				267	
	2	−1086.344	2198.687	2245.322	2204.104	0.908	0.24	<0.001	27 (11%); 240 (89%)	0.907–0.981
	**3**	−**1036.084**	**2108.169**	**2172.739**	**2115.669**	**0.787**	**<0.05**	**<0.001**	**17 (6%); 145 (54%); 105 (40%)**	**0.896**–**0.967**
	4	−1009.311	2064.622	2147.129	2074.205	0.916	0.25	<0.001	9 (3%); 30 (12%); 133 (49%); 95 (36%)	0.920–0.969
	5	−981.724	2019.448	2119.891	2031.115	0.874	0.17	<0.001	10 (4%); 37 (14%); 102 (37%); 24 (9%); 94 (36%)	0.865–0.978
	6	−956.331	1978.662	2097.042	1992.412	0.860	<0.05	<0.001	10 (4%); 37 (14%); 102 (37%); 43 (17%); 51 (19%); 24 (9%)	0.862–0.979
SWI non-mbg	1	−378.109	772.218	792.392	767.140				92	
	2	−339.743	705.486	738.270	697.234	0.864	0.11	<0.001	18 (20%); 74 (80%)	0.901–0.973
	**3**	−**327.833**	**691.666**	**737.058**	**680.240**	**0.804**	**0.27**	**<0.001**	**40 (43%); 42 (46%); 10 (11%)**	**0.847–0.966**
	4	−315.268	676.537	734.538	661.937	0.879	0.65	<0.001	9 (11%); 50 (53%); 30 (33%); 3 (3%)	0.905–0.996
	5	−305.499	666.998	737.608	649.225	0.897	0.10	<0.001	3 (3%); 3 (3%); 9 (11%); 37 (41%); 40 (41%)	0.897–1.000
	6	−298.301	662.603	745.822	641.656	0.888	0.67	0.36	3 (3%); 3 (3%); 29 (32%); 38 (39%); 14 (15%); 5 (7%)	0.906–0.996

Classification accuracy relates to the average latent class probabilities. Model fit for the chosen profile solution is in bold

*SWI* Switzerland, nat national sample, *mbg* migration background sample, *non-mbg* non-migration background sample, *AIC* Akaike information criterion, *BIC* Bayesian information criterion, *aBIC* Sample-size adjusted BIC, *aLMR LRT* Lo-Mendell-Rubin adjusted likelihood ratio test, *BLRT* Bootstrap likelihood ratio test

## Footnotes

1 McDonald’s omega ω were also calculated as reliability coefficients and were equal to the Cronbach alphas (or differed by 0.01 in one case).
