# Supplementary material for: Minority and Majority Adolescents’ Attitudes toward Mutual Acculturation and its Association with Psychological Adjustment
Source: J Youth Adolesc. 2022 Apr 6;51(8):1511–35. doi: 10.1007/s10964-022-01604-6 (PMC9232442; doi:10.1007/s10964-022-01604-6)
Supplement: Supplementary file 1 — Supplementary Materials [file 10964_2022_1604_MOESM1_ESM.docx]

# **Online Supplementary Material**

## Items Verbatim

**Table 1B***Assessment of Attitudes Toward Mutual Acculturation: Items Verbatim (Sidler et al., 2021)*

| Dimension | Migration background students’ heritage culture maintenance | Migration background students’ dominant culture adoption | Majority students’ acquisition of cultural knowledge | Schools’ endorsement of intercultural contact |
| --- | --- | --- | --- | --- |
| Introduction | I find that it is important for teenagers from another country who live in [country] … | | I find it is important that [country] teenagers who live in [country] have to get to know… | I find it is important that the [country] schooling system allows teenagers from other countries and [country] teenagers… |
| Item 1 | …to be allowed to preserve their traditions and customs. | …to adopt the dominant traditions and customs in [country]. | … the traditions and customs of teenagers from other countries who live in [country]. | … to exchange information about their traditions and customs. |
| Item 2 | …to be allowed to preserve their religion. | …to adopt the dominant religion in [country]. | … the religions of teenagers from other countries who live in [country]. | … to exchange information about their religions. |
| Item 3 | …to be allowed to preserve their way of life. | …to adopt the dominant way of life in [country]. | … the ways of life of teenagers from other countries who live in [country]. | … to exchange information about their ways of life. |
| Item 4 | …to be allowed to preserve their language. | …to adopt (one of) the official language in [country]. | … the languages of teenagers from other countries who live in [country]. | … to exchange information about their languages. |
| Item 5 | …to be allowed to preserve their way of clothing. | …to adopt the dominant way of clothing in [country]. | … the ways of clothing of teenagers from other countries who live in [country]. | … to exchange information about their ways of clothing. |
| Item 6 | …to be allowed to preserve their family culture. | …to adopt the dominant family culture in [country]. | … the family cultures of teenagers from other countries who live in [country]. | … to exchange information about their family cultures. |
| Item 7 | …to be allowed to preserve their views on professional careers of women and men. | …to adopt the dominant views on professional careers of women and men in [country]. | … the views on professional careers of women and men of teenagers from other countries who live in [country]. | … to exchange information about their views on professional careers of women and men. |
|  | | | | |

## Confirmatory Factor Analysis

The original 28-item and four-factor model was tested through confirmatory factor analyses (CFA) in JASP (0.16.0.0) for each country. Results showed sufficient fit (Xia & Yang, 2019) in the German sample (χ^2^_(344)_ = 647.557, p < 0.001; RMSEA = .062 [90% CI = .055–.070]; SRMR = .058; CFI = .910; TLI = .901), the Greek sample (χ^2^_(344)_ = 799.608, p < 0.001; RMSEA = .067 [90% CI = .061–.073]; SRMR = .050; CFI = .905; TLI = .896), and the Swiss sample (χ^2^_(344)_ = 755.626, p < 0.001; RMSEA = .067 [90% CI = .061–.074]; SRMR = .049; CFI = .916; TLI = .907).

**Table 2B***Confirmatory Factor Analyses of the Four Acculturation Dimensions in Germany, Greece, and Switzerland*

| Dimension | Items | | Germany | | | | Greece | | | | Switzerland | | | |
| --- | --- | --- | --- | --- | --- | --- | --- | --- | --- | --- | --- | --- | --- | --- |
|  |  | Factor | 1 | 2 | 3 | 4 | 1 | 2 | 3 | 4 | 1 | 2 | 3 | 4 |
| Adolescents from other countries should be allowed to maintain their… | traditions | | .60 |  |  |  | .67 |  |  |  | .63 |  |  |  |
|  | religion | | .66 |  |  |  | .79 |  |  |  | .72 |  |  |  |
|  | way of life | | .73 |  |  |  | .66 |  |  |  | .68 |  |  |  |
|  | language | | .68 |  |  |  | .69 |  |  |  | .68 |  |  |  |
|  | way of clothing | | .67 |  |  |  | .80 |  |  |  | .74 |  |  |  |
|  | family culture | | .74 |  |  |  | .63 |  |  |  | .60 |  |  |  |
|  | gendered views on careers | | .60 |  |  |  | .55 |  |  |  | .56 |  |  |  |
| Adolescents from other countries should adopt the dominant … in [country]. | traditions | |  | .83 |  |  |  | .80 |  |  |  | .87 |  |  |
|  | religion | |  | .76 |  |  |  | .76 |  |  |  | .80 |  |  |
|  | way of life | |  | .77 |  |  |  | .77 |  |  |  | .81 |  |  |
|  | language | |  | .62 |  |  |  | .65 |  |  |  | .62 |  |  |
|  | way of clothing | |  | .81 |  |  |  | .81 |  |  |  | .89 |  |  |
|  | family culture | |  | .79 |  |  |  | .78 |  |  |  | .87 |  |  |
|  | gendered views on careers | |  | .59 |  |  |  | .59 |  |  |  | .66 |  |  |
| [country] adolescents should get to know … of adolescents from other countries living in [country]. | traditions | |  |  | .82 |  |  |  | .80 |  |  |  | .87 |  |
|  | religions | |  |  | .71 |  |  |  | .74 |  |  |  | .81 |  |
|  | ways of life | |  |  | .83 |  |  |  | .87 |  |  |  | .86 |  |
|  | languages | |  |  | .61 |  |  |  | .70 |  |  |  | .70 |  |
|  | ways of clothing | |  |  | .76 |  |  |  | .78 |  |  |  | .76 |  |
|  | family cultures | |  |  | .79 |  |  |  | .81 |  |  |  | .79 |  |
|  | gendered views on careers | |  |  | .77 |  |  |  | .77 |  |  |  | .81 |  |
| [country] schools should make it possible for all adolescents to exchange information about … | traditions | |  |  |  | .77 |  |  |  | .76 |  |  |  | .83 |
|  | religions | |  |  |  | .73 |  |  |  | .80 |  |  |  | .78 |
|  | ways of life | |  |  |  | .80 |  |  |  | .79 |  |  |  | .82 |
|  | languages | |  |  |  | .74 |  |  |  | .75 |  |  |  | .78 |
|  | ways of clothing | |  |  |  | .77 |  |  |  | .71 |  |  |  | .71 |
|  | family cultures | |  |  |  | .69 |  |  |  | .75 |  |  |  | .79 |
|  | gendered views of careers | |  |  |  | .75 |  |  |  | .74 |  |  |  | .78 |

## Measurement Invariance Across Countries

To assess measurement invariance across countries, a multigroup confirmatory factor analysis was run in JASP (0.16.0.0) with country as the grouping variable.

**Table 3B***Multigroup Confirmatory Factor Analysis to Assess Measurement Invariance Across Countries (MG-CFA MI).*

| Model | Type of test | Compared with | χ^2^ | *df* | *RMSEA* | *CFI* | *TLI* | *SRMR* | Δdf | Δ*CFI* | Δ*RMSEA* | Δ*SRMR* | Decision |
| --- | --- | --- | --- | --- | --- | --- | --- | --- | --- | --- | --- | --- | --- |
| M1a | Germany |  | 647.557 *p* < .001 | 344 | .062 [.055, .070] | .910 | .901 | .058 |  |  |  |  |  |
| M1b | Greece |  | 799.608 *p* < .001 | 344 | .067 [.061, .073] | .905 | .896 | .050 |  |  |  |  |  |
| M1c | Switzerland |  | 755.626 *p* < .001 | 344 | .067 [.061, .074] | .916 | .907 | .049 |  |  |  |  |  |
| M2 | Configural invariance |  | 2258.490 *p* < .001 | 1088 | .064 [.060, .068] | .910 | .907 | .060 |  |  |  |  |  |
| M3 | Metric invariance | M2 | 2251.443 *p* < .001 | 1080 | .064 [.060, .068] | .910 | .906 | .056 | –8 | 0 | 0 | –.004 | Accept |
| M4 | Scalar invariance | M3 | 2361.079 *p* < .001 | 1128 | .064 [.061, .068] | .906 | .905 | .055 | 148 | –.004 | 0 | –.001 | Accept |
| M5 | Strict invariance | M4 | 2512.122  *p* < .001 | 1184 | .065 [.062, .069] | .898 | .903 | .057 | 56 | –.008 | .001 | .002 | Accept |
| *Note*. χ^2^, chi-square statistics; df, degrees of freedom; RMSEA, root mean square error of approximation; CFI, comparative fit index; TLI, Tucker Lewis index; SRMR, standardized root mean square error of approximation; Δ, change in statistical values. | | | | | | | | | | | | | |
